# GiSAO.db: a database for ageing research

**DOI:** 10.1186/1471-2164-12-262

**Published:** 2011-05-24

**Authors:** Edith Hofer, Gerhard T Laschober, Matthias Hackl, Gerhard G Thallinger, Günter Lepperdinger, Johannes Grillari, Pidder Jansen-Dürr, Zlatko Trajanoski

**Affiliations:** 1 Institute for Genomics and Bioinformatics, Graz University of Technology, Petersgasse 14, 8010 Graz, Austria; 2 Division for Bioinformatics, Biocenter, Innsbruck Medical University, Schöpfstrasse 45, 6020 Innsbruck, Austria; 3 Institute for Biomedical Aging Research, Austrian Academy of Sciences, Rennweg 10, 6020 Innsbruck, Austria; 4 Aging and Immortalization Research, Department of Biotechnology, University of Natural Resources and Applied Life Sciences Vienna, Muthgasse 18, 1190 Vienna, Austria

## Abstract

**Background:**

Age-related gene expression patterns of *Homo sapiens *as well as of model organisms such as *Mus musculus*, *Saccharomyces cerevisiae*, *Caenorhabditis elegans *and *Drosophila melanogaster *are a basis for understanding the genetic mechanisms of ageing. For an effective analysis and interpretation of expression profiles it is necessary to store and manage huge amounts of data in an organized way, so that these data can be accessed and processed easily.

**Description:**

GiSAO.db (Genes involved in senescence, apoptosis and oxidative stress database) is a web-based database system for storing and retrieving ageing-related experimental data. Expression data of genes and miRNAs, annotation data like gene identifiers and GO terms, orthologs data and data of follow-up experiments are stored in the database. A user-friendly web application provides access to the stored data. KEGG pathways were incorporated and links to external databases augment the information in GiSAO.db. Search functions facilitate retrieval of data which can also be exported for further processing.

**Conclusions:**

We have developed a centralized database that is very well suited for the management of data for ageing research. The database can be accessed at https://gisao.genome.tugraz.at and all the stored data can be viewed with a guest account.

## Background

Accumulated cell damage is one of the main perpetrators of ageing. The damage is caused by a variety of different factors and conditions, including somatic mutations, mitochondrial dysfunction and oxidative stress [1,2]. If damage of cellular components (proteins, nucleic acids, lipids, etc.) remains permanently and is not corrected by repair systems (e.g. DNA repair or the elimination of damaged organelles and proteins), then cellular senescence and/or apoptosis is occuring. Cellular senescence and apoptosis contribute to a characteristic ageing phenotype as well as to the development of age-related diseases [3,4]. Since the underlying mechanisms of cellular ageing, leading to senescence or perhaps to apoptosis, have not yet been fully revealed, it is indispensable to identify and study genes and miRNAs which are involved in the ageing process. An effective way to determine these genes and gene-regulatory miRNAs are genome-wide studies of expression patterns, as it is well known that expression profiles of organisms change with age [2,5]. Microarrays are well suited for this task as they are a high-throughput method for determining the expression of tens of thousands of genes in parallel [6]. Results of microarray experiments are usually validated by applying low-throughput methods for measuring gene expression, e.g. qPCR or Northern blots [7], or confirmed with protein assays like Western blots.

Genetic research into human ageing is supported by investigation of ageing in various model organisms, such as *Mus musculus, Saccharomyces cerevisiae, Caenorhabditis elegans *and *Drosophila melanogaster*. These organisms have a much shorter lifespan than humans and can be easily genetically manipulated for experimental purposes. The results obtained from model organisms can be transferred to a certain extent to *Homo sapiens*, since these organisms share orthologous genes [8]. However, in order to effectively analyse data generated in various experiments using different organisms, it is necessary to structure and manage this data in an organized way.

Therefore, several publicly available databases which store ageing specific gene information were developed: the Human Aging Genomic Resources (HAGR) [9], the Gene Aging Nexus (GAN) [10], the Aging Gene Database [11], the Atlas of Gene Expression in Mouse Aging Project (AGEMAP) [12], and the NetAge database [13]. However, to the best of our knowledge, there is no database which contains microarray gene expression data together with orthologous genes, ageing-related microarray miRNA expression data as well as data of follow-up experiments. We have therefore initiated the development of a database GiSAO.db (**G**enes **i**nvolved in **s**enescence, **a**poptosis and **o**xidative stress) to support ongoing and future studies in experimental ageing research.

## Construction and content

**Figure 1:**
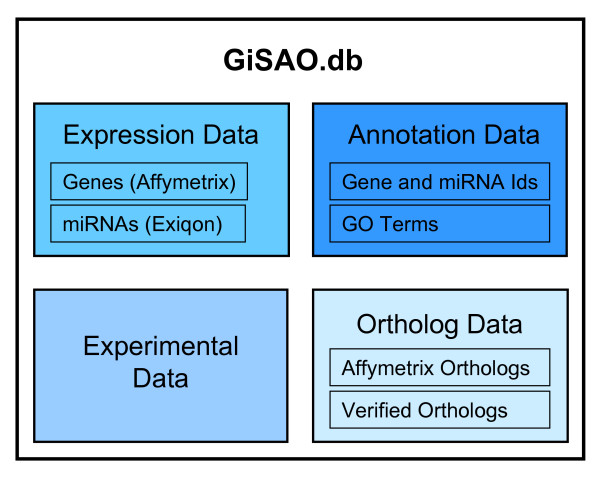
**GiSAO overview**. Four different types of data are stored in the database: gene expression data obtained from Affymetrix microarray experiments and miRNA expression data from Exiqon microarray experiments, annotations for genes and miRNAs including GO terms, orthologs data provided by Affymetrix as well as manually entered verified orthologs data and experimental data of follow-up experiments.

GiSAO.db is a database for storing and managing expression data of genes involved in senescence, apoptosis and oxidative stress. It is connected to a web application which provides an easy and controlled access to this data. Specifically, the database is capable of storing four data types: expression data, annotation data, orthologous data and data of follow-up experiments (Figure 1).

### Expression data

Normalized gene and miRNA expression values obtained from microarray experiments investigating ageing reside in GiSAO.db. It is possible to store gene expression data from Affymetrix one-colour microarrays as well as miRNA expression data from Exiqon two-colour microarrays in the database.

### Annotation data

For genes, several gene identifiers are available as annotation in GiSAO.db: GeneSymbol, Refseq Id, Gene Name, EntrezGene Id, UniProt Id, UniGene Id, SGD Id, MGI Id, FlyBase Id, RGD Id and AGI Id. These identifiers were obtained together with Gene Ontology (GO) [14] terms from Affymetrix which provides annotation data of each spot on a microarray chip [15]. In case of miRNAs, the miRNA name and the miRBase Id [16] are stored as annotations in the database.

### Orthologs data

Two types of orthologous data are included in GiSAO.db: orthologs provided by Affymetrix and verified orthologs data. Pairs of orthologs probe sets from different Affymetrix microarray chips are stored. Moreover, verified orthologous gene pairs between different species retrieved from other sources, such as literature or orthologs databases can be entered manually.

### Experimental data

Finally, GiSAO.db provides facilities to store data of follow-up experiments. An experiment is specified by properties such as type (e.g. qPCR, Western blot), classification (e.g. senescence, inflammation), organism and cell type. All genes that were investigated can be linked to the experiment and antibodies or primers can be defined. Moreover, it is possible to specify references, and protocols as well as result files may be uploaded and attached to experiments or their associated genes.

### Database content

GiSAO.db provides annotation data for five Affymetrix microarrays: Human Genome U133 Plus 2.0 Array, Mouse Genome 430 2.0 Array, Yeast Genome 2.0 Array, C. elegans Genome Array and Drosophila Genome Array. Furthermore, annotations for two custom made human Exiqon miRNA microarrays are available. The database contains orthologs provided by Affymetrix between *Homo sapiens, Mus musculus, Saccharomyces cerevisiae, Caenorhabditis elegans *and *Drosophila melanogaster*. Currently GiSAO.db stores gene expression values of 11 experiments comprising 111 Affymetrix microarrays of three different species: *Homo sapiens, Mus musculus *and *Saccharomyces cerevisae*. Additionally there are 7 human miRNA experiments with 40 Exiqon microarrays stored in the database. Moreover, numerous verified orthologs and data of several follow-up experiments are available in GiSAO.db.

Normalization of expression data obtained from Affymetrix microarray experiments was performed using the gcrma algorithm [17] in CARMAweb [18]. Independently of the particular experiments, data of a certain cell type, e.g. HUVEC or PFF, were normalized together.

The statistical framework R [19] and the Bioconductor package limma [20] were used for background correction of miRNA expression data with the normexp algorithm as well as lowess normalization [21].

### Implementation

The GiSAO.db database system was developed using the object-oriented and platform independent Java programming language [22]. Based on the Java Enterprise Edition (Java EE) platform [23], a three-tier application composed of a relational database, business logic and presentation layer was implemented.

In order to control data access and manage user data, the web application offers an integrated authentication and authorization system [24].

## Utility

### User Interface

**Figure 2:**
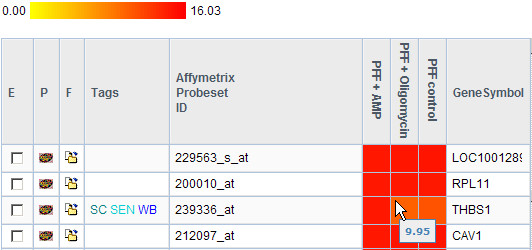
**Display of expression values obtained from Affymetrix one-colour arrays**. The values are colour coded to facilitate the identification of prominent expression values and comparison between values of a gene of different arrays. Additionally, tags are displayed for a quick overview of experimental data. In this case, for the gene THBS a follow-up experiment was performed with the organism *Homo sapiens *(HS), experiment classification *oxidative stress *(OS) and experiment type *qPCR *(QP).

The database system offers a user-friendly web interface which facilitates data input and retrieval. Results of microarray experiments can be viewed in detail as both the expression value of each spot for one-colour microarray chips, and the expression ratio between the colour channels for two-colour microarrays are displayed. Expression values and ratios are represented by colour-coded boxes which facilitate the determination of highly expressed genes, up- or down-regulated miRNAs and the comparison of expression values and ratios of different microarrays (Figure 2). The values and ratios can be displayed in a logarithmic or decimal scale, and a threshold can be defined to show only those genes or miRNAs whose expression values or ratios exceed the defined cut-off.

**Figure 3:**
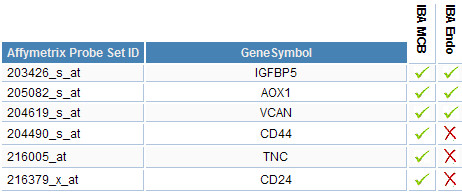
**Favourite gene list comparison**. Genes of interest can be collected in lists and two or more favourite lists can be compared to determine common genes.

Pairs of orthologous genes can be retrieved using a simple search function which returns Affymetrix orthologs as well as verified orthologs. For experimental data of follow-up experiments, the application provides a flexible query mechanism which accepts organism, experiment classification and experiment type as parameters. Additionally, tags are displayed in gene lists to provide basic information about the different experiments performed on a gene at first glance. Tags are essentially shortcuts describing experiment classification, experiment type and organism, referencing experimental data (Figure 2). Genes or miRNAs which are of special interest for users can be assembled to *favourite lists *and furnished with additional information. These lists can be compared to check for common entries (Figure 3).

**Figure 4:**
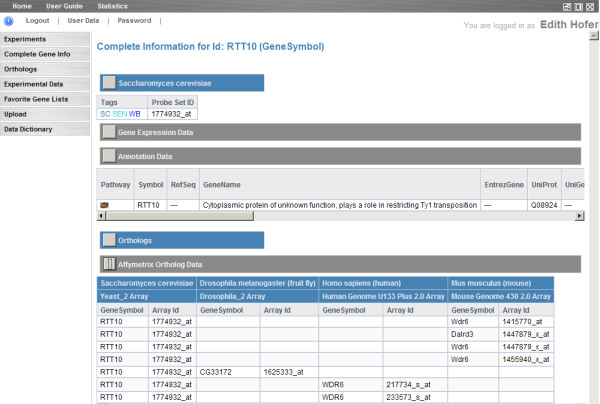
**Gene search result**. For the queried gene all the information stored in the database, i.e. tags, gene expression data, annotation data and orthologs data are listed.

A comprehensive search function that takes gene and miRNA Ids as parameters provides access to all data about a gene or miRNA in GiSAO.db. The search yields expression data, annotation data, orthologs, experimental data tags and favourite lists of the specified gene or miRNA (Figure 4). An export mechanism which enables further processing of data from the database in external tools is seamlessly included into GiSAO.db. Lists of expression values, favorite genes and orthologs can be written to plain text files, PDF files or files in comma separated values (CSV) format.

Identifiers of genes and miRNAs as well as GO terms are provided to attribute a meaning to the probe (set) identifiers of the microarray spots. To enhance stored components with additional information, links to external databases are offered. Gene Ids are linked to their respective databases, e.g. RefSeq, Entrez Gene or UniProt, and to the ortholog databases HomoloGene [25] and InParanoid [26]. Moreover, pathways from the Kyoto Encyclopedia of Genes and Genomes (KEGG) [27] can be accessed in GiSAO.db via a web service provided by KEGG.

### Data Submission

There are two ways of entering data in the database: manual data input using web forms or data upload from files. Forms are enhanced by a data dictionary concept, which extends the functionality of select fields, facilitates data input, and prevents inconsistencies in the database caused by spelling mistakes or duplicate entries.

Verified orthologs and experimental data can be entered in GiSAO.db using specific upload forms. Experimental data can be additionally uploaded from files as well as favorite lists, expression data, annotation data and Affymetrix ortholog data to accelerate the data input process. Affymetrix and Exiqon annotation data of microarray chips as well as Affymetrix ortholog data are updated on a regular basis by the producers. To adopt these changes in GiSAO.db, update functions have been implemented.

Moreover, several protocols or result files of follow-up experiments can be uploaded at once by using a Java applet. For each file upload, a feedback report is created which allows the user to check whether the upload was successful and view error messages in case something went wrong.

### Authentication and authorization

To protect the data in the database fine grained access rights were defined in the integrated authentication and authorization system [24]. Three different user roles can access GiSAO.db: administrator, user and guest. An "administrator" can add, edit and delete all data. A "user" is allowed to add data, edit and delete his/her own data or data belonging to a member of the user's institute. Finally, a user assigned the "guest" role may view all the data stored in the database but has no rights to add, edit or delete any data.

### Case study

Expression data of 47 human Affymetrix microarrays stored in GiSAO.db were analyzed. The experiments were performed on different cell types and were divided into two different groups: premature senescence induced by oxidative stress and other senescence models. The derived expression profiles were compared to determine conserved patterns in the various cellular aging models. As a result 484 genes associated with oxidative stress-induced senescence, 1087 genes associated with other senescence models, and 155 genes which seem to play a role in both experimental settings were determined [28]. This information guided the selection of 93 candidate genes which were tested for their ability to modulate lifespan in a unicellular model system (yeast chronological lifespan) to study organismic ageing [28]. Thereby, several new pathways which may be important in cellular senescence were identified [28]. Additionally, GiSAO.db was used supporting another study investigating the contribution of miRNAs in ageing [21].

Up to date 87 expression data sets stored in GiSAO.db have also been published in the public repository ArrayExpress [29]: 51 Affymetrix arrays from 6 experiments (E-MEXP-2283, E-MEXP-2285, E-MEXP-2167, E-MEXP-2345, E-MEXP-1506 and E-MEXP-2683) as well as 36 Exiqon arrays from 6 experiments (E-MEXP-2386, E-MEXP-2425, E-MEXP-2393, E-MEXP-2398, E-MEXP-2455, E-MEXP-2459).

## Discussion

GiSAO.db is a database for the storage and management of ageing-related data. The core of GiSAO.db consists of normalized gene expression and miRNA expression data retrieved from microarray experiments. Annotation data like gene or miRNA identifiers as well as GO terms are available to interpret the expression profiles. As many of the orthologs provided by Affymetrix are only predicted ones, a manual curation of verified orthologs was implemented. The orthologs in the database facilitate cross-species comparison of expression profiles and the detection of evolutionary conserved expression patterns. Data of follow-up experiments, e.g. qPCR or Western blot experiments complement the microarray expression data. For a quick overview on these follow-up experiments performed for a specific gene, tags which serve as links to experimental data can be added.

Additionally, links to external gene, miRNA and ortholog databases are offered as well as KEGG pathways. Data upload and update is performed asynchronously, meaning that GISAO.db can be used while the upload takes place. In web forms data dictionary fields support controlled, yet customizable data input to keep the database content consistent. Search functions deliver data from the database which can then be exported in various file formats that are suitable for direct import in programs like MS Excel that are used for further processing of the data.

Furthermore, genes or miRNAs of interest can be grouped into favorite lists which can then be compared among different research groups. A sophisticated authentication and authorization system prevents undesired manipulation of data, yet allows all users to view the entire content of the database. By using a three-tier architecture, maintenance and extension of the application is facilitated and the various layers, e.g. the underlying database system, may also be exchanged. The usability of the web application is greatly enhanced by Web 2.0 functionality which was added using AJAX technology.

## Conclusions

We have developed GiSAO.db, a system for storage and management of ageing-related gene data. An intuitive user interface provides fast and organised access to these data. Additionally, references to external databases are offered to elaborate the data in the database. These features in combination with the stored gene and miRNA expression data, annotation data, orthologs data and data of follow-up experiments make it a powerful tool for genetic ageing research.

## Availability

GiSAO.db is available at http://gisao.genome.tugraz.at. All the data stored in the database can be viewed with a guest account. Username and password for guest users are provided on the login page of the application.

## Authors' contributions

EH designed and implemented the application and drafted the manuscript. GGT contributed to conception, design, and implementation of the application. PJD contributed to the concept of the database, was involved in data upload and data quality control issues, and contributed to writing the manuscript. GL, MH, and GL contributed to the concept of the database and were involved in data upload. PJD and ZT were responsible for the overall project coordination. All authors gave final approval of the version to be published.
